# Sequencing conjugated polymers by eye

**DOI:** 10.1126/sciadv.aas9543

**Published:** 2018-06-15

**Authors:** Daniel A. Warr, Luís M. A. Perdigão, Harry Pinfold, Jonathan Blohm, David Stringer, Anastasia Leventis, Hugo Bronstein, Alessandro Troisi, Giovanni Costantini

**Affiliations:** 1 Department of Chemistry, University of Warwick, Coventry CV4 7AL, UK.; 2 Department of Chemistry, Imperial College London, London SW7 2AZ, UK.; 3 Department of Chemistry, University of Cambridge, Cambridge CB2 1EW, UK.; 4 Department of Chemistry, University of Liverpool, Liverpool L69 7ZD, UK.

## Abstract

The solid-state microstructure of a conjugated polymer is the most important parameter determining its properties and performance in (opto)-electronic devices. A huge amount of research has been dedicated to tuning and understanding how the sequence of monomers, the nature and frequency of defects, the exact backbone conformation, and the assembly and crystallinity of conjugated polymers affect their basic photophysics and charge transporting properties. However, because of the lack of reliable high-resolution analytical techniques, all the structure-property relations proposed in the literature are based either on molecular modeling or on indirect experimental data averaged on polydisperse samples. We show that a combination of electrospray vacuum deposition and high-resolution scanning tunneling microscopy allows the imaging of individual conjugated polymers with unprecedented detail, thereby unraveling structural and self-assembly characteristics that have so far been impossible to determine.

## Teaser

Electrospray deposition and STM as a new analytical technique to sequence conjugated polymers and reveal polymerization defects.

## INTRODUCTION

The steadily increasing research in conjugated polymers is motivated by these materials being at the heart of low-cost, lightweight, and flexible (opto)-electronic applications such as organic photovoltaics, light-emitting diodes, transistors, sensors, actuators, and supercapacitors. In recent years, it has emerged that the electronic properties of these materials (such as charge carrier mobility, energy band gap, and adsorption spectrum) are controlled at their most fundamental level by six main characteristics of the polymer microstructure: (i) the polymer chemical composition, (ii) the planarity of the backbone (*1*, *2*), (iii) the relative conformation of the heterocycles (*3*), (iv) the conformation and interdigitation of the solubilizing side chains (*4*), (v) the interaction between polymer strands (*5*), and (vi) the presence and nature of chemical defects such as regioregularity (*6*, *7*) or polymerization defects (*8*–*10*).

The precise microstructure of conjugated polymers is, however, very difficult to determine, in particular for the new generation of materials that are based on complex compositions of monomers obtained through multi- and copolymerization techniques. Here, the control on the final product is reliant upon differences in kinetic reaction rates, with side reactions being more difficult to prevent and the intrinsic statistical nature of the polymerization process becoming more apparent. Polymer sizes and mass distributions are mainly evaluated by chromatography [for example, size exclusion chromatography (SEC)] and mass spectroscopy (for example, matrix-assisted laser desorption/ionization), although these techniques often struggle to handle the complexity and heterogeneity of last-generation conjugated polymers. In particular, questions on the presence and nature of polymerization defects and on what the exact monomer sequence within a copolymer is remain essentially unanswered. This constitutes a major limitation to further progress in the field, as it hampers the possibility of better understanding the polymerization process and thus achieving a more precise control of the ensuing functional materials.

Here, we propose a novel and radically different approach to the analytics of conjugated polymers, based on high-resolution scanning tunneling microscopy (STM). To fully harness the analytical power of STM and to image individual molecular species with subnanometer resolution, it is essential that the molecules are deposited in vacuum onto atomically clean and flat surfaces and that the measurements are performed in situ. We achieve this by exploiting recent advances in the soft landing of thermolabile molecules (*11*, *12*), thereby demonstrating a significant development in vacuum electrospray deposition (ESD) (*13*–*17*). Here, we report the combination of ESD with submolecular-resolution STM to analyze diketopyrrolopyrrole (DPP)–based polymers. We demonstrate the ability to identify the monomer units and the solubilizing alkyl side chains and use this to precisely sequence the polymer structure. We show that we can determine the nature, locate the position, and ascertain the number of defects in the polymer backbone. Our analysis also reveals that the main driver for backbone conformation of surface-adsorbed polymers is the maximization of alkyl side-chain interdigitation, which leads to unexpected cis conformations of the heterocycles. This unique insight into the microstructure of conjugated polymers is not attainable by any other existing analytical technique and represents a fundamental advancement in polymer analytics.

## RESULTS

**Fig. 1:**
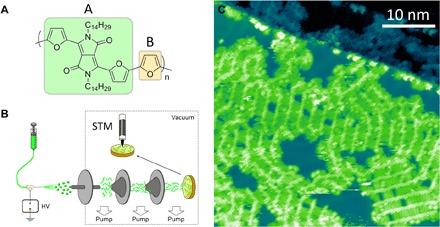
Vacuum deposition and STM imaging of C_14_DPPF-F polymers. (**A**) Molecular structure of C_14_DPPF-F. (**B**) Schematic representation of the experimental setup. HV, high voltage. (**C**) STM image showing C_14_DPPF-F adsorbed on Au(111) after annealing to 100°C. The polymer backbones appear as bright rows, and the alkyl side chains are seen as darker rows perpendicular to the backbones. *V*_bias_ = −1.8 V, *I* = 300 pA.

Poly(tetradecyl-diketopyrrolopyrrole-furan-*co*-furan) (C_14_DPPF-F; Fig. 1A) is a conjugated polymer of the DPP-based family (*18*) that is currently showing some of the best performance in optoelectronic devices. Figure 1C shows a typical STM image of C_14_DPPF-F deposited by electrospray on a Au(111) surface at room temperature and annealed to 100°C, demonstrating that both the polymer backbone and the alkyl side chains can be clearly identified. The backbones of the polymers are mostly straight and tightly aligned with the [$11\bar{2}$] directions of the Au(111) herringbone reconstruction, even bending around the elbow sites of the reconstruction, which indicates a strong molecule-substrate interaction. The alkyl chains are generally oriented perpendicular to the backbone and are thus aligned along the [$\bar{1}10$] substrate directions. The polymer strands self-assemble into extended two-dimensional (2D) islands through attractive interactions between the alkyl side chains. Chains of neighboring polymer strands interdigitate to maximize van der Waals contact, resulting in an interstrand separation of 2.6 ± 0.1 nm, in excellent agreement with that observed for small furan-DPP molecules (*19*). Thin-film x-ray diffraction (XRD; see fig. S1) showed a lamellar stacking distance of ~2 nm, which is considerably shorter than the total width of the molecule (~4 nm), indicating that interdigitation is likely to be occurring also in the solid state [as has been observed for other conjugated polymers (*4*)]. Therefore, we speculate that the intermolecular ordering observed here provides insight into the local assembly of a bulk sample.

**Fig. 2:**
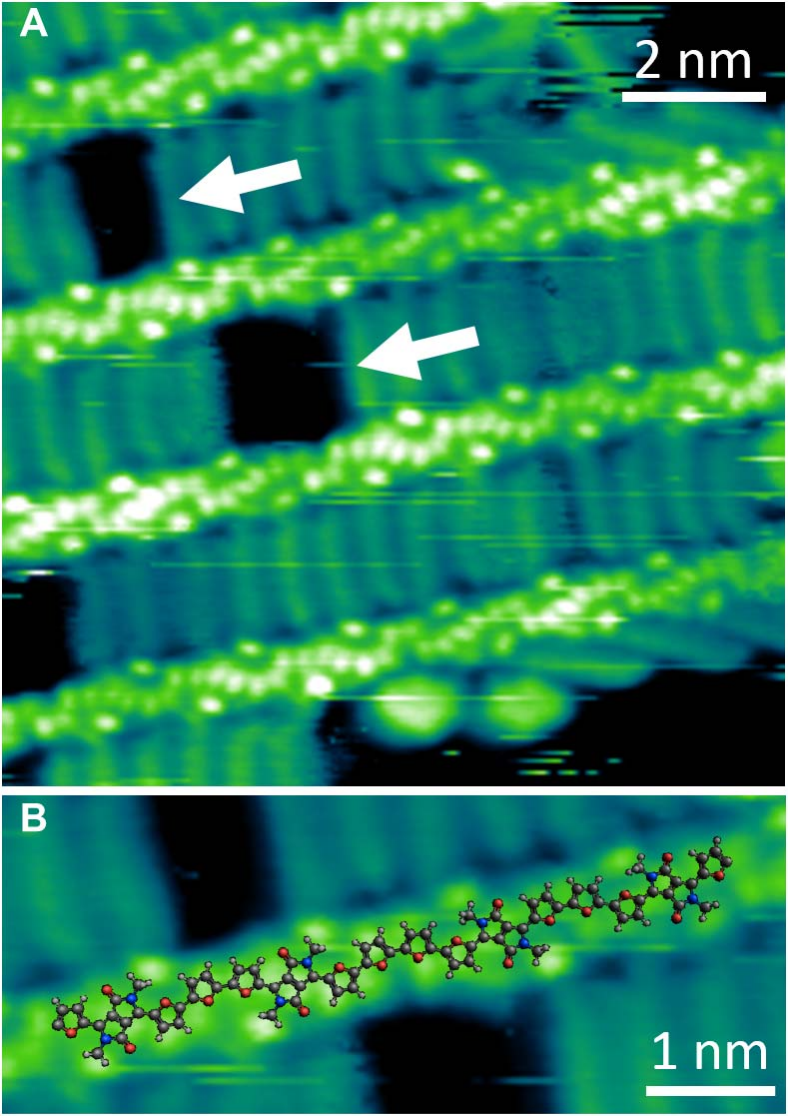
High-resolution STM images of C_14_DPPF-F polymers on Au(111). (**A**) Submolecular resolution of the polymer backbone and the interdigitation of the alkyl side chains. White arrows indicate gaps in the alkyl chain interdigitation. (**B**) Molecular model of the polymer backbone overlaid on a section of C_14_DPPF-F (C atoms are shown in gray, O in red, N in blue, and H in white). The alkyl chains have been substituted with CH_3_ groups for better visualization. An ABBA defect is visible in the center of the image. *V*_bias_ = −1.8 V, I = 300 pA.

Higher-magnification STM images (Fig. 2) display a periodic submolecular contrast, allowing the identification of the individual monomer units—DPPF (A) and furan (B)—and their sequence in the polymer chain. To assign the features appearing in the backbone, the unambiguous location of the alkyl side chains is used as a reference. The chains extend from two circular lobes situated at opposite positions with respect to the main polymer axis (Fig. 2B). Quantum chemistry calculations show that the alkyl chains are not coplanar with the DPP moiety (fig. S2), allowing us to identify these two lobes with the protruding start of the alkyl chains and to precisely locate the A monomer. This corresponds to four aligned bright dots within the backbone, two central ones for the DPP unit, and two on each side for the lateral furan rings (fig. S4B). The DPPs form an angle of about ±45° with respect to the straight backbone axis. The position of the B monomer is also determined by using a geometry-optimized molecular model (see fig. S4C) and appears to coincide with a fifth bright dot. The so-determined AB repeat unit has an excellent match with any STM image containing a section of polymer with no defects.

**Fig. 3:**
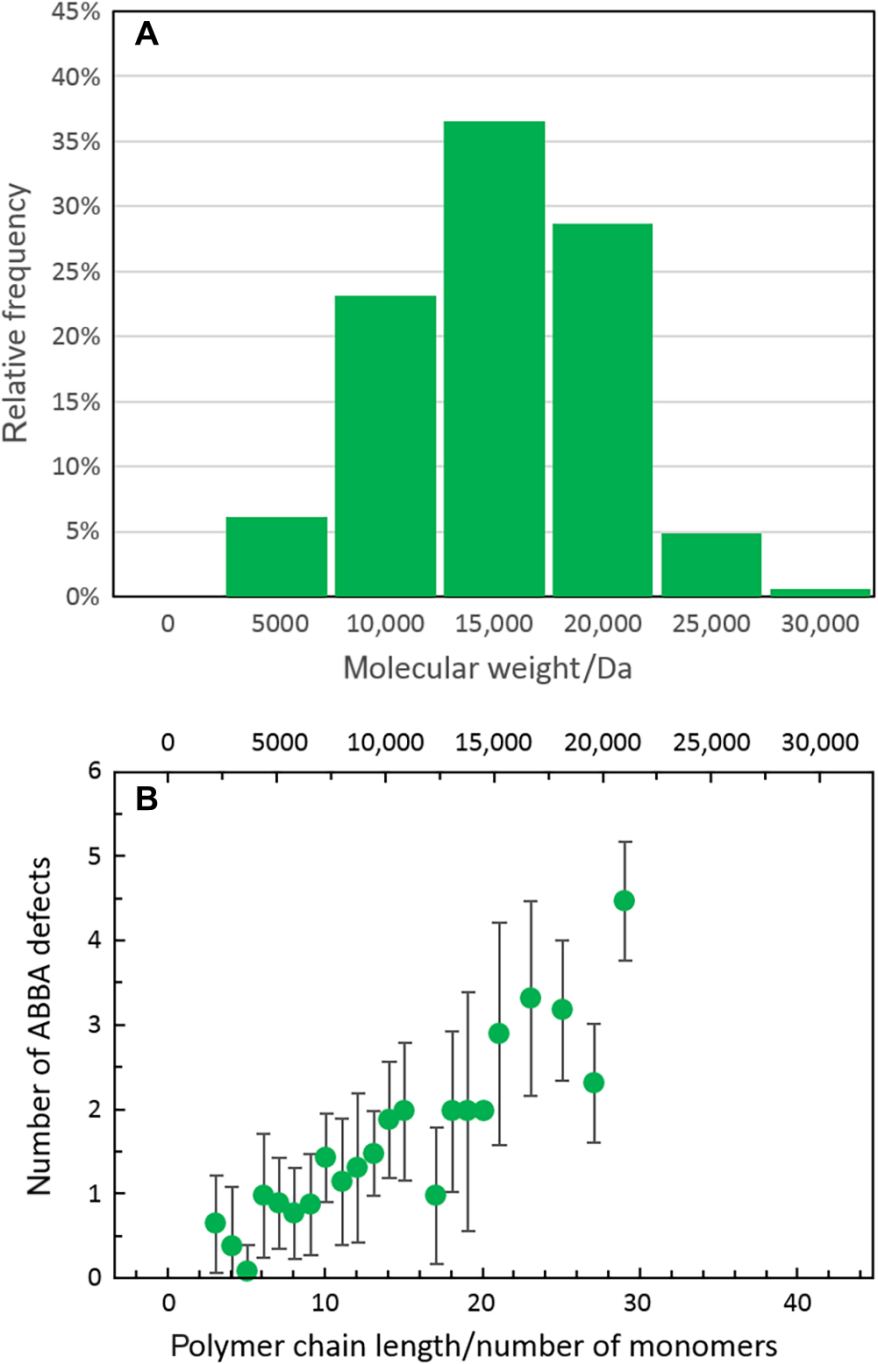
Analysis of the mass distribution and defect frequency of C_14_DPPF-F. (**A**) Histogram of molecular weight distribution determined from STM images (see text for detailed methodology). (**B**) Frequency of ABBA defects as a function of the polymer chain length expressed as number of (AB) monomers or molecular weight. A linear dependence is visible.

By employing this molecular model, the STM images can be used to evaluate the mass distribution of the polymer chains. The number of repeat units is obtained by measuring the length of a large number of polymer strands (164), resulting in an average value of 17 ± 7. By multiplying this by the mass of a single repeat unit (724 Da), the average mass of the polymers is evaluated to be 12.2 ± 4.8 kDa (the full size and mass distribution are shown in Fig. 3A). This value can be compared with what is obtained from SEC measurements on the same polymer, which resulted in a number average (*M*_n_) and weight average (*M*_w_) molecular mass of 6.4 and 37.3 kDa, respectively (as measured by high-temperature SEC; figs. S5 and S6). The mismatch in the measured molecular weights is likely a consequence of the methodology used. SEC is known to provide inaccurate evaluations of the true molecular weight of conjugated polymers (*20*), as they are measured relative to polystyrene standards that have notably different hydrodynamic radii, with uncertainties up to a factor of 2 having been reported (*21*). In addition, solution-aggregated species are commonly observed in narrow band-gap conjugated polymers (*22*), further affecting the measured molecular weight.

Two main types of defects can be recognized in the 2D assembly of C_14_DPPF-F: darker gaps in the interdigitation sequence of alkyl side chains and circular protrusions, which can be found either within these gaps or at the edge of polymer islands (Fig. 2A). While the circular protrusions are attributed to impurities either in the solvent or in the sample, the gaps are linked to defects in the monomer sequence of the polymer. Each gap is accompanied by a change in the orientation of the submolecular features in one of the two polymer backbones delimiting the gap itself (wider gaps, as in the center of Fig. 2A, show an inversion in both flanking polymer backbones). Moreover, an extra bright dot is always observed between successive DPP units in correspondence to the gaps. While in the regular parts of the polymer strand the distance between consecutive DPPs is 1.4 ± 0.1 nm—corresponding well to the expected periodicity of a regular (AB)_n_ polymer sequence—it becomes 1.8 ± 0.1 nm across the gaps.

The defective regions can be explained by the same molecular model described in fig. S4C if, at the position of the gaps, the model is inverted through a mirror plane perpendicular to the polymer backbone, resulting in an extra furan ring and an ABBA monomer sequence (Fig. 2B). A detailed analysis of the high-resolution STM images can thus reveal the presence of defects in the monomer sequence and identifies these as ABBA (instead of the regular ABAB) arrangements. Only this type of defect was observed in all analyzed images, with the exception of two single occurrences of an ABBBA defect. An evaluation of a large number of polymer strands (180) shows that there is approximately one defect in every 10 nm of strand length, which is equivalent to one extra B monomer for every 8.5 AB units. In particular, Fig. 3B shows that the number of defects in a polymer strand scales linearly with its length, as would be expected for a random inclusion of defects. Moreover, Fig. 3B also demonstrates that the ESD-STM technique does not preferentially image chains with greater or smaller defect concentrations.

**Fig. 4:**
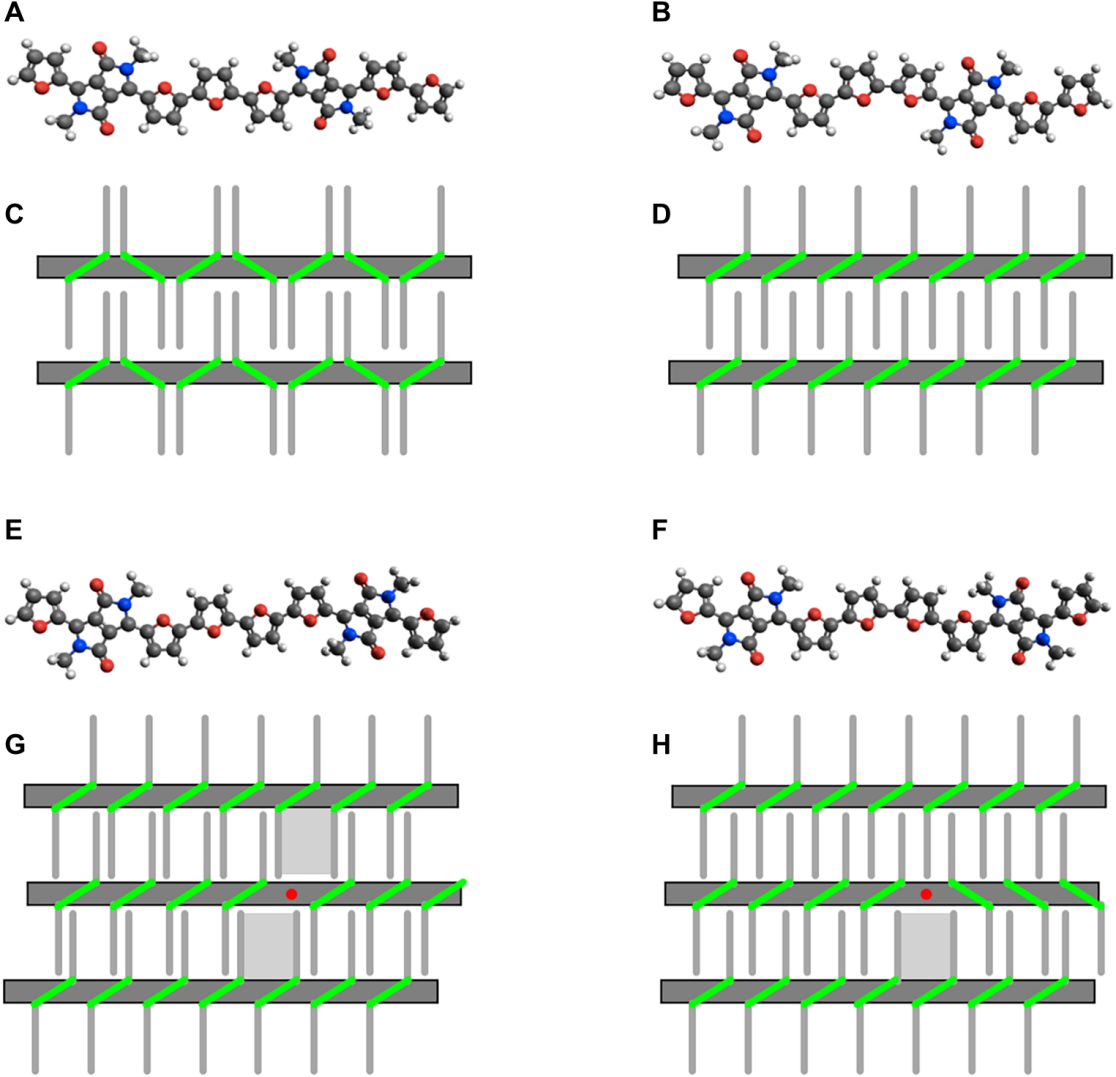
Molecular structure and intermolecular interactions of pristine and defective C_14_DPPF-F polymers. (**A** and **B**) Structure of defect-free C_14_DPPF-F in the all-trans configuration (A) and with a single furan-furan cis arrangement (B), demonstrating specular and parallel DPP orientations, respectively. (**C** and **D**) Schematic representation of interstrand interactions for the polymer configurations corresponding to (A) and (B), respectively. The alkyl chains are represented by thin gray lines, and the DPP units are represented by green segments. (**E** and **F**) Structure of C_14_DPPF-F around an ABBA defect in the all-trans configuration (E) and with a single furan-furan cis arrangement (F). The DPP units across the defect are arranged in a parallel and specular orientation, respectively. (**G** and **H**) Schematic representation of interstrand interactions for the polymer configurations corresponding to (E) and (F), respectively. The ABBA defects are represented by red dots, and larger gaps in the chain interdigitation are represented by gray-shaded areas.

It is evident from the STM images that the polymer side-chain interaction, maximized by interdigitation, strongly influences the orientation of the alkyl chains. This is seen in Fig. 2A, where a number of chains at the edge of a molecular island (top and bottom right corner of Fig. 2A) are significantly distorted with respect to those within the island. Perhaps more surprisingly, the drive to maximize alkyl chain interactions is also responsible for the orientation of the monomers within the polymer backbone. While the commonly assumed all-trans conformation of the furan units predicts an alternating orientation of the DPPs (Fig. 4A), the STM images show that the DPP units are all parallel to each other in the defect-free regions of the polymer (Fig. 2). Alternating orientations of the DPP moieties imply alternating large and small separations between the alkyl chains, which are not ideal for intermolecular interactions (Fig. 4C). On the other hand, a backbone configuration, where one of the furan units of the A monomer is cis to the furan of the B monomer (Fig. 4B), would have equally spaced alkyl chains, allowing an ideal interdigitation (Fig. 4D). The conformation in Fig. 4B is obtained from that in Fig. 4A through 180° rotations around the C–C bonds between the A and B monomers and is thus characterized by having all DPP units parallel to each other, as is experimentally observed. Density functional theory (DFT) calculations show that the conformation in Fig. 4B is only 0.06 eV less stable than that in Fig. 4A and that the two can easily interconvert through a barrier of 0.32 eV (fig. S3).

The parallel orientation of the DPPs is disrupted across ABBA defects, where the DPP units are specular to each other (Fig. 2). Also in this case, optimization of the intermolecular interaction is the driving force because an all-trans conformation with an extra furan ring (parallel DPPs; see Fig. 4E) would create two gaps in the side-chain interdigitation, one on each side of the defective strand (Fig. 4G). On the contrary, the experimentally observed configuration, obtained by a 180° rotation around the C–C bond connecting the two central furans (specular DPPs; Fig. 4F), generates a gap only on one side of the defective strand and thus allows a better alkyl chain interdigitation (Fig. 4H).

It should be noticed that the backbone of a defect-free isolated C_14_DPPF-F polymer in the conformation with parallel DPPs would not be straight but curved, due to the optimal angle between heterocycles (see vacuum DFT-optimized structure in fig. S3). However, this curved conformation is observed only in isolated strands while, in the majority of cases, the polymer backbones are straight within the molecular islands (fig. S7). This latter conformation maximizes side-chain interdigitation by having evenly spaced alkyl chains, and the calculations show that the energetic cost of straightening the polymer backbone is compensated by the better alkyl-alkyl chain interactions formed in ideally interdigitated monolayers (fig. S3). The strong polymer-substrate interaction and the likely electronic hybridization between the two are expected to play an important role, too. On the other hand, the phenomena presented here do not depend on the specific metallic substrate, because the same alkyl chain interdigitation and the same structure of the backbone (straightness, monomer orientation, and defects) were observed also when C_14_DPPF-F was deposited on Ag(111) (figs. S7B and S8).

## DISCUSSION

In summary, we have presented a radical new approach to polymer analytics based on the concept of structural analysis through high-resolution microscopy (*23*). The unprecedented spatial resolution of our STM images allows us to precisely sequence conjugated polymers by simply counting the monomer units. This is used to demonstrate the presence of unexpected ABBA defects in the C_14_DPPF-F copolymer and to quantify their occurrence. The existence of excitonic and electronic trap states in a conjugated polymer backbone has long been discussed, and their origin has often been loosely described as impurities in the form of morphological or chemical defects (*24*, *25*). Little was known about the nature and the frequency of defects, although it has always been assumed that they were either torsional defects along the backbone—for example, hairpins (*26*)—or chemical defects—such as homo- or mis-couplings (*27*, *28*). In the specific case of DPP polymers, it has been suggested that the primary chemical defects result from the homocoupling between the DPP-containing units (*9*, *10*). Here, we show unambiguously that this is not always the case by identifying the chemical defects in the C_14_DPPF-F copolymer as homocouplings between furan rings. By proving the possibility of attaining a detailed understanding of the amount and types of defects in conjugated polymers, our work demonstrates the potential to finally resolve this long-standing issue and to establish an essential—presently still missing—structure-property relationship. A further emerging field of research that would highly benefit from the analytical advances demonstrated here is the recent development of greener methods for the synthesis of conjugated polymers. New synthetic strategies such as the direct arylation polymerization (*29*) produce polymers with often inferior characteristics to those obtained using conventional reaction schemes [for example, via the Stille cross-coupling reaction (*30*)]. Although progress has been made using the conventional analytical techniques of nuclear magnetic resonance and mass spectrometry, it is still not clear what the origin of the defects is and how they can be avoided. Using the ESD-STM technique, it would be possible to analyze polymers synthesized by different methods, identify and quantify the defects, and then relate these to the measured optical and electronic properties.

The analysis of the STM data also allowed us to determine the orientation of individual monomers within the polymer backbone, showing that surface-adsorbed polymers adopt unexpected conformations to optimize intermolecular interactions. These results represent a further unique insight into the microstructure of conjugated polymers that is not attainable by any other existing analytical technique.

Because the sequence of a polymer is not altered when adsorbed on a substrate, the chemical composition uniquely determined from our STM analysis is valid in absolute terms and thus relevant to any type of device or application involving this material. This might not be true for some aspects of the observed 2D assembly, which could result from the interaction with a metallic surface and thus not be representative of the solid-state bulk packing. However, we believe that other features—such as the maximization of alkyl chain interdigitation—are very general and that our work thus lends insight relevant for the local packing of functional polymeric thin films.

The combination of vacuum ESD and STM appears to be a quite generally applicable technique, as we have recently successfully tested it on a number of different π-conjugated polymers (for example, fig. S9). We speculate that this novel approach might have profound impact on the wider field of polymer science, representing a first fundamental step in tackling a major and still unresolved problem, that is, how to precisely and reliably characterize a polymeric macromolecule with monomeric precision.

## MATERIALS AND METHODS

Details of the synthesis of the C_14_DPPF-F polymer are reported in the Supplementary Materials. A solution of C_14_DPPF-F was prepared at a concentration of 40 μg/ml in a 3:1 mixture by volume of toluene/methanol. Au(111) and Ag(111) on mica substrates (Georg Albert PVD) were cleaned in ultrahigh vacuum through repeated cycles of Ar^+^ sputtering (1 keV, 3 μA/cm^2^) and subsequent annealing at 500°C for 20 min. The surface was checked for atomic flatness and cleanliness with in situ STM measurements before each polymer deposition. The C_14_DPPF-F polymer was deposited for 15 min by ESD in vacuum (Molecularspray Ltd.) onto the Au(111) substrate at a pressure of 1 × 10^−7^ mbar. After deposition, the pressure in the preparation chamber reverted to its base value of 4 × 10^−10^ mbar, and the sample was annealed to 100°C. The sample was then transferred without breaking the vacuum to the analysis chamber (base pressure, <2 × 10^−10^ mbar) and imaged by STM.

STM images were acquired in the constant-current mode, using an electrochemically etched tungsten tip. All voltages were applied to the sample with respect to the tip. All images in the paper and the Supplementary Materials were acquired at a sample temperature of −153°C. Images acquired at room temperature show the same structures but appear noisier, probably due to enhanced surface diffusion of the molecular species. Gwyddion 2.44 software (*31*) was used to process the STM images.

## Supplementary Material

http://advances.sciencemag.org/cgi/content/full/4/6/eaas9543/DC1

## SUPPLEMENTARY MATERIALS

Supplementary material for this article is available at http://advances.sciencemag.org/cgi/content/full/4/6/eaas9543/DC1 section S1. Synthesis of the C_14_DPPF-F polymer section S2. XRD data of C_14_DPPF-F thin films section S3. Conformation energy difference in model system via ab initio calculations section S4. Assignment of A and B units in submonomeric resolved STM images of C_14_DPPF-F section S5. SEC analysis of C_14_DPPF-F section S6. Surface-adsorbed C_14_DPPF-F polymer strands section S7. Preliminary ESD-STM data on PDPPTPT fig. S1. XRD of a drop cast thin film of C_14_DPPF-F. fig. S2. Ab initio calculations of the conformation of alkyl chains with respect to the polymer backbone. fig. S3. Gas-phase optimized structure of a C_14_DPPF-F oligomer. fig. S4. High-resolution STM images of C_14_DPPF-F and corresponding molecular models. fig. S5. GPC molecular weight analysis of C_14_DPPF-F at 80°C. fig. S6. GPC molecular weight analysis of C_14_DPPF-F at 160°C. fig. S7. STM images of C_14_DPPF-F polymers deposited on Au(111) and Ag(111). fig. S8. STM images of C_14_DPPF-F polymers deposited on Ag(111) after annealing to 100°C. fig. S9. STM images of PDPPTPT polymers deposited on Au(111) after annealing to 100°C. References (*32*–*36*)

## References

[1] Zhang X., Bronstein H., Kronemeijer A. J., Smith J., Kim Y., Kline R. J., Richter L. J., Anthopoulos T. D., Sirringhaus H., Song K., Heeney M., Zhang W., McCulloch I., DeLongchamp D. M., Molecular origin of high field-effect mobility in an indacenodithiophene-benzothiadiazole copolymer. Nat. Commun. 4, 2238 (2013). 23900027 10.1038/ncomms3238

[2] Venkateshvaran D., Nikolka M., Sadhanala A., Lemaur V., Zelazny M., Kepa M., Hurhangee M., Kronemeijer A. J., Pecunia V., Nasrallah I., Romanov I., Broch K., McCulloch I., Emin D., Olivier Y., Cornil J., Beljonne D., Sirringhaus H., Approaching disorder-free transport in high-mobility conjugated polymers. Nature 515, 384–388 (2014). 25383522 10.1038/nature13854

[3] Vezie M. S., Few S., Meager I., Pieridou G., Dörling B., Ashraf R. S., Goñi A. R., Bronstein H., McCulloch I., Hayes S. C., Campoy-Quiles M., Nelson J., Exploring the origin of high optical absorption in conjugated polymers. Nat. Mater. 15, 746–753 (2016). 27183327 10.1038/nmat4645

[4] McCulloch I., Heeney M., Bailey C., Genevicius K., MacDonald I., Shkunov M., Sparrowe D., Tierney S., Wagner R., Zhang W., Chabinyc M. L., Kline R. J., McGehee M. D., Toney M. F., Liquid-crystalline semiconducting polymers with high charge-carrier mobility. Nat. Mater. 5, 328–333 (2006). 16547518 10.1038/nmat1612

[5] Noriega R., Rivnay J., Vandewal K., Koch F. P. V., Stingelin N., Smith P., Toney M. F., Salleo A., A general relationship between disorder, aggregation and charge transport in conjugated polymers. Nat. Mater. 12, 1038–1044 (2013). 23913173 10.1038/nmat3722

[6] Kim Y., Cook S., Tuladhar S. M., Choulis S. A., Nelson J., Durrant J. R., Bradley D. D. C., Giles M., McCulloch I., Ha C.-S., Ree M., A strong regioregularity effect in self-organizing conjugated polymer films and high-efficiency polythiophene: Fullerene solar cells. Nat. Mater. 5, 197–203 (2006).

[7] Ying L., Huang F., Bazan G. C., Regioregular narrow-bandgap-conjugated polymers for plastic electronics. Nat. Commun. 8, 14047 (2017). 28348399 10.1038/ncomms14047 PMC5379056

[8] Hu D., Yu J., Wong K., Bagchi B., Rossky P. J., Barbara P. F., Collapse of stiff conjugated polymers with chemical defects into ordered, cylindrical conformations. Nature 405, 1030–1033 (2000). 10890438 10.1038/35016520

[9] Hendriks K. H., Li W., Heintges G. H. L., van Pruissen G. W. P., Wienk M. M., Janssen R. A. J., Homocoupling defects in diketopyrrolopyrrole-based copolymers and their effect on photovoltaic performance. J. Am. Chem. Soc. 136, 11128–11133 (2014). 25029494 10.1021/ja505574a

[10] Hong W., Chen S., Sun B., Arnould M. A., Meng Y., Li Y., Is a polymer semiconductor having a “perfect” regular structure desirable for organic thin film transistors? Chem. Sci. 6, 3225–3235 (2015). 29142690 10.1039/c5sc00843c PMC5657407

[11] Rader H. J., Rouhanipour A., Talarico A. M., Palermo V., Samorì P., Müllen K., Processing of giant graphene molecules by soft-landing mass spectrometry. Nat. Mater. 5, 276–280 (2006). 16532002 10.1038/nmat1597

[12] Rauschenbach S., Vogelgesang R., Malinowski N., Gerlach J. W., Benyoucef M., Costantini G., Deng Z., Thontasen N., Kern K., Electrospray ion beam deposition: Soft-landing and fragmentation of functional molecules at solid surfaces. ACS Nano 3, 2901–2910 (2009). 19775085 10.1021/nn900022p

[13] Rauschenbach S., Ternes M., Harnau L., Kern K., Mass spectrometry as a preparative tool for the surface science of large molecules. Annu. Rev. Anal. Chem. 9, 473–498 (2016). 10.1146/annurev-anchem-071015-041633 27089378

[14] O’Sullivan M. C., Sprafke J. K., Kondratuk D. V., Rinfray C., Claridge T. D. W., Saywell A., Blunt M. O., O’Shea J. N., Beton P. H., Malfois M., Anderson H. L., Vernier templating and synthesis of a 12-porphyrin nano-ring. Nature 469, 72–75 (2011). 21209660 10.1038/nature09683

[15] Jethwa S. J., Madsen M., Knudsen J. B., Lammich L., Gothelf K. V., Linderoth T. R., Revealing the structural detail of individual polymers using a combination of electrospray deposition and UHV-STM. Chem. Commun. 53, 1168–1171 (2017). 10.1039/c6cc09167a 28054080

[16] Yokoyama T., Kogure Y., Kawasaki M., Tanaka S., Aoshima K., Scanning tunneling microscopy imaging of long oligothiophene wires deposited on Au(111) using electrospray ionization. J. Phys. Chem. C 117, 18484–18487 (2013).

[17] Förster S., Widdra W., Structure of single polythiophene molecules on Au (001) prepared by in situ UHV electrospray deposition. J. Chem. Phys. 141, 054713 (2014). 25106606 10.1063/1.4891929

[18] Nielsen C. B., Turbiez M., McCulloch I., Recent advances in the development of semiconducting DPP-containing polymers for transistor applications. Adv. Mater. 25, 1859–1880 (2013). 23008141 10.1002/adma.201201795

[19] Fu C., Bélanger-Gariépy F., Perepichka D. F., Supramolecular ordering of difuryldiketopyrrolopyrrole: The effect of alkyl chains and inter-ring twisting. CrstEngComm 18, 4285–4289 (2016).

[20] Kreyenschmidt M., Uckert F., Muellen K., A new soluble poly (p-phenylene) with tetrahydropyrene repeating units. Macromolecules 28, 4577–4582 (1995).

[21] Hayashi S., Yamamoto S.-i., Koizumi T., Effects of molecular weight on the optical and electrochemical properties of EDOT-based π-conjugated polymers. Sci. Rep. 7, 1078 (2017). 28439088 10.1038/s41598-017-01132-5 PMC5430904

[22] Bijleveld J. C., Gevaerts V. S., Di Nuzzo D., Turbiez M., Mathijssen S. G. J., de Leeuw D. M., Wienk M. M., Janssen R. A. J., Efficient solar cells based on an easily accessible diketopyrrolopyrrole polymer. Adv. Mater. 22, E242–E246 (2010). 20734379 10.1002/adma.201001449

[23] Feynman R. P., There’s plenty of room at the bottom. Eng. Sci. 23, 22–36 (1960).

[24] Baniya S., Khanal D., Lafalce E., You W., Vardeny Z. V., Optical studies of native defects in π-conjugated donor–acceptor copolymers. J. Appl. Phys. 123, 161571 (2018).

[25] Mikhnenko O. V., Blom P. W. M., Nguyen T.-Q., Exciton diffusion in organic semiconductors. Energ. Environ. Sci. 8, 1867–1888 (2015).

[26] Zhang W., Milner S. T., Gomez E. D., Nematic order imposes molecular weight effect on charge transport in conjugated polymers. ACS Cent. Sci. 4, 413–421 (2018). 29632888 10.1021/acscentsci.8b00011 PMC5879482

[27] Lee J., Kalin A. J., Yuan T., Al-Hashimi M., Fang L., Fully conjugated ladder polymers. Chem. Sci. 8, 2503–2521 (2017). 28553483 10.1039/c7sc00154a PMC5431637

[28] T. C. Parker, S. R. Marder, *Synthetic Methods in Organic Electronic and Photonic Materials: A Practical Guide* (Royal Society of Chemistry, 2015).

[29] Bura T., Blaskovits J. T., Leclerc M., Direct (hetero)arylation polymerization: Trends and perspectives. J. Am. Chem. Soc. 138, 10056–10071 (2016). 27463826 10.1021/jacs.6b06237

[30] Bura T., Beaupré S., Légaré M.-A., Quinn J., Rochette E., Blaskovits J. T., Fontaine F.-G., Pron A., Li Y., Leclerc M., Direct heteroarylation polymerization: Guidelines for defect-free conjugated polymers. Chem. Sci. 8, 3913–3925 (2017). 28966781 10.1039/c7sc00589j PMC5578375

[31] Nečas D., Klapetek P., Gwyddion: An open-source software for SPM data analysis. Cent. Eur. J. Phys. 10, 181–188 (2012).

[32] Hirono Y., Kobayashi K., Yonemoto M., Kondo Y., Metal-free deprotonative functionalization of heteroaromatics using organic superbase catalyst. Chem. Commun. 46, 7623–7624 (2010). 10.1039/c0cc03106b 20848038

[33] Bijleveld J. C., Karsten B. P., Mathijssen S. G. J., Wienk M. M., de Leeuw D. M., Janssen R. A. J., Small band gap copolymers based on furan and diketopyrrolopyrrole for field-effect transistors and photovoltaic cells. J. Mater. Chem. 21, 1600–1606 (2011).

[34] Yiu A. T., Beaujuge P. M., Lee O. P., Woo C. H., Toney M. F., Fréchet J. M. J., Side-chain tunability of furan-containing low-band-gap polymers provides control of structural order in efficient solar cells. J. Am. Chem. Soc. 134, 2180–2185 (2012). 22191680 10.1021/ja2089662

[35] Troisi A., Shaw A., Very large π-conjugation despite strong nonplanarity: A path for designing new semiconducting polymers. J. Phys. Chem. Lett. 7, 4689–4694 (2016). 27806576 10.1021/acs.jpclett.6b02367

[36] Chickos J. S., Acree W. E. Jr, Enthalpies of vaporization of organic and organometallic compounds, 1880–2002. J. Phys. Chem. Ref. Data 32, 519–878 (2003).

