# Supplementary material for: Sequencing conjugated polymers by eye
Source: Sci Adv. 2018 Jun 15;4(6):eaas9543. doi: 10.1126/sciadv.aas9543 (PMC6003723; doi:10.1126/sciadv.aas9543)
Supplement: http://advances.sciencemag.org/cgi/content/full/4/6/eaas9543/DC1 [file supp_4_6_eaas9543__index.html]

Science Advances | Science Advances

## Supplementary Materials

**This PDF file includes:**

- section S1. Synthesis of the C14DPPF-F polymer
- section S2. XRD data of C14DPPF-F thin films
- section S3. Conformation energy difference in model system via ab initio calculations
- section S4. Assignment of A and B units in submonomeric resolved STM images of C14DPPF-F
- section S5. SEC analysis of C14DPPF-F
- section S6. Surface-adsorbed C14DPPF-F polymer strands
- section S7. Preliminary ESD-STM data on PDPPTPT
- fig. S1. XRD of a drop cast thin film of C14DPPF-F.
- fig. S2. Ab initio calculations of the conformation of alkyl chains with respect to the polymer backbone.
- fig. S3. Gas-phase optimized structure of a C14DPPF-F oligomer.
- fig. S4. High-resolution STM images of C14DPPF-F and corresponding molecular models.
- fig. S5. GPC molecular weight analysis of C14DPPF-F at 80°C.
- fig. S6. GPC molecular weight analysis of C14DPPF-F at 160°C.
- fig. S7. STM images of C14DPPF-F polymers deposited on Au(111) and Ag(111).
- fig. S8. STM images of C14DPPF-F polymers deposited on Ag(111) after annealing to 100°C.
- fig. S9. STM images of PDPPTPT polymers deposited on Au(111) after annealing to 100°C.
- References (*32–36*)

Download PDF

**Files in this Data Supplement:**

- Adobe PDF - aas9543\_SM.pdf
