# Supplementary material for: Sequencing conjugated polymers by eye
Source: Sci Adv. 2018 Jun 15;4(6):eaas9543. doi: 10.1126/sciadv.aas9543 (PMC6003723; doi:10.1126/sciadv.aas9543)
Supplement: http://advances.sciencemag.org/cgi/content/full/4/6/eaas9543/DC1 [file aas9543_SM.pdf]

Science Advances

AAAS

advances.sciencemag.org/cgi/content/full/4/6/eaas9543/DC1

Supplementary Materials for

Sequencing conjugated polymers by eye

Daniel A. Warr, Luís M. A. Perdigão, Harry Pinfold, Jonathan Blohm, David Stringer, Anastasia Leventis, Hugo Bronstein, Alessandro Troisi, Giovanni Costantini

Published 15 June 2018, *Sci. Adv.* **4**, eaas9543 (2018)
DOI: 10.1126/sciadv.aas9543

This PDF file includes:

- section S1. Synthesis of the C_{14}DPPF-F polymer
- section S2. XRD data of C_{14}DPPF-F thin films
- section S3. Conformation energy difference in model system via ab initio calculations
- section S4. Assignment of A and B units in submonomeric resolved STM images of C_{14}DPPF-F
- section S5. SEC analysis of C_{14}DPPF-F
- section S6. Surface-adsorbed C_{14}DPPF-F polymer strands
- section S7. Preliminary ESD-STM data on PDPPTPT
- fig. S1. XRD of a drop cast thin film of C_{14}DPPF-F.
- fig. S2. Ab initio calculations of the conformation of alkyl chains with respect to the polymer backbone.
- fig. S3. Gas-phase optimized structure of a C_{14}DPPF-F oligomer.
- fig. S4. High-resolution STM images of C_{14}DPPF-F and corresponding molecular models.
- fig. S5. GPC molecular weight analysis of C_{14}DPPF-F at 80°C.
- fig. S6. GPC molecular weight analysis of C_{14}DPPF-F at 160°C.
- fig. S7. STM images of C_{14}DPPF-F polymers deposited on Au(111) and Ag(111).
- fig. S8. STM images of C_{14}DPPF-F polymers deposited on Ag(111) after annealing to 100°C.
- fig. S9. STM images of PDPPTPT polymers deposited on Au(111) after annealing to 100°C.
- References (*32*–*36*)

Supplementary Materials

section S1. Synthesis of the C_{14}DPPF-F polymer

General experimental information

All reactions were carried out in a fume hood under argon conditions unless specified in the synthetic procedure and stirred using a magnetic plate and stir bar. Anhydrous solvents were removed under argon and all starting reagents were used as supplied. Column chromatography and silica plugs were performed using Geduran silica gel 60 (40-63 μm) and thin-layer chromatography was carried out using DC Fertigfolien ALUGRAM aluminium sheets coated in silica gel. UV Visible absorption spectroscopy was recorded using a Shimadzu UV-1800 Spectrophotometer. ^{1}H NMR spectra were recorded at 300 MHz on a Bruker AMX300 spectrometer and 400 MHz on a Bruker Advance III 400 spectrometer in the solvent stated. Shifts are reported to the nearest 0.01ppm and defined with the abbreviations: s, singlet; d, doublet; t, triplet; qn, quintet; sxt, sextet; m, multiplet. Coupling constants (J) are measured in Hertz. ^{13}C NMR spectra was recorded on a Bruker Advance III 400 spectrometer in deuterated chloroform with shifts reported to the nearest 0.1 ppm. Mass spectra were obtained at the UCL Chemistry Mass Spectrometry Facility. High temperature (160° C) GPC/SEC Samples were run at the Warwick Polymer Characterisation Research Technology Platform on an Agilent PL220 instrument equipped with differential refractive index (DRI) and viscometry (VS) detectors. The system was equipped with 2 x PLgel Olexis columns (2*300 x 7.5 mm) and a PLgel Olexis guard column. The mobile phase was 1,2,4-trichlorobenzene (TCB) with 250 PPM BHT (butylated hydroxytoluene) additive. Samples were run at 1 ml/min at 160 ^{o}C. Polystyrene standards (Agilent EasyVials) were used to create a third order calibration. Analyte samples were filtered through a stainless steel frit with 10 μm pore size before injection. Respectively, experimental molar mass (M_n, SEC) and dispersity (Đ) values of synthesized polymers were determined by conventional calibration using Agilent GPC/SEC software. Low temperature (80° C) GPC/SEC sample were run on a Shimadzu Prominence instrument equipped with a differential refractive index and a UV-Vis detector. The system was equipped with 2 x Phenomenex Phenogel 10 μm 300 x 7.8mm linear columns and a Phenogel 10 μm guard column. The mobile phase was chlorobenzene (CB). Samples were run at 2 mL/min at 80 ^{o}C. Polystyrene standards (Agilent EasyVials) were used to create a third order calibration. Analyte samples were filtered through a PTFE frit with 10 μm pore size before injection. Respectively, experimental molar mass (M_n, SEC) and dispersity (Đ) values of synthesized polymers were determined by conventional calibration using Shimadzu GPC/SEC software.

2-Furonitrile (*32*): Iodine beads (30.00g, 118.20 mmol) were added to a stirring solution of ammonia water (600mL) and 2-furaldehyde (11.00g, 114.48 mmol) open to the air. THF (90 mL) was added and the reaction mixture stirred at RT for 10 min after which saturated sodium sulphite solution (100 mL) was added and the layers separated. The aqueous phase was then extracted with diethyl ether (3x150mL) and the organic fractions dried over Mg_2SO_4, and concentrated in vacuo to yield the crude product as a brown oil (8.87g, 83%) which was used directly in the next step. **^{1}H NMR** (300 MHz, $CDCl_3$) δ (ppm): 7.59 (d, J = 2.0 Hz, 1H), 7.11 (d, J = 3.6 Hz, 1H), 6.54 (dd, J = 3.6, 1.8 Hz, 1H). **LRMS** (EI+) *m/z*: 93.

3,6-bis(furan-2-yl)2,5-dihydropyrrolo[3,4-c]pyrrole-1,4-dione (*33*): Sodium chunks (0.89g, 38.61 mmol) were added to a three-neck RBF containing 1-methyl-2-butanol (20.5 mL). Iron (III) chloride (25mg) was then added and the reaction stirred for 1hr at 100°C before the addition of 2-furonitrile (2.50g, 26.86mmol) and diethyl succinate (2.44g, 13.99mmol) in 2-methyl-2-butanol (6mL). The reaction mixture was stirred at 110°C for 2 hr before being allowed to cool to 55°C and quenched with methanol (33.5mL) and glacial acetic acid (10mL). The quenched reaction was stirred for 20hr at 55°C before the solid was isolated by filtration. This was then washed with water (20 mL), methanol (3x20 mL), hexane (20 mL) and finally acetone (20 mL) to yield a pink solid (2.34g, 65%). **^{1}H NMR** (400 MHz, DMSO-d6)

δ (ppm): 11.18 (s, 2H), 8.05 (d, J = 1.7, 0.7 Hz, 2H), 7.66 (dd, J = 3.6, 0.6Hz, 2H), 6.84 (dd, J = 3.6, 1.7 Hz, 2H). **MALDI-TOF** (*m/z*): 270.

1-Bromotetradecane: Triphenyl phosphine (17.50g, 66.67mmol) and 1-tetradecanol (11.51g, 53.67mmol) were dissolved in stirring dichloromethane (50 mL) in a 100 mL RBF that was open to air. The reaction mixture was cooled to 0°C and N-bromosuccinimide (11.87g, 66.67mmol) added portion-wise over 5 min. The ice bath was removed and the reaction stirred at RT for 24hr. The solution was diluted further with DCM (30mL) and then quenched with water (50mL). The organic layer was then separated, dried over Mg_2SO_4 and concentrated *in vacuo*. Flash column chromatography (hexane) afforded the product as a colourless oil (6.09g, 41%). **^{1}H NMR** (300 MHz, $CDCl_3$) δ (ppm): 3.40 (t, J = 6.9 Hz, 2H), 1.85 (qn, J = 7.5 Hz, 2H), 1.49-1.16 (m, 22H), 0.89 (t, J = 6.4 Hz, 3H). **LRMS** (EI+) *m/z*: 276.

2,5-bis(tetradecyl)-3,6-di(furan-2-yl)2,5-dihydropyrrolo[3,4-c] pyrrole-1,4-dione (*33*): 3,6-bis(furan-2-yl)2,5-dihydropyrrolo[3,4-c]pyrrole-1,4-dione (0.55g, 2.05mmol), 1-bromotetradecane (1.71g, 6.15mmol), 18-crown-6 (0.25g) and K_2CO_3 (0.85g, 6.15mmol) were added to a 250mL three-neck RBF. Anhydrous DMF (55mL) was added and the reaction vigorously stirred for 18 hr at 120°C. The reaction was then allowed to cool to room temperature and the solvents removed *in vacuo*. The resulting solid was washed with cold hexane (20 mL) and then dissolved in chloroform and passed through a silica plug. Addition of methanol (50 mL) and filtration yielded the product as a pink solid (0.913g, 67%). Rf = 0.3 (hexane/chloroform 2:1). **^{1}H NMR** (400 MHz, $CDCl_3$): δ 8.31 (d, J = 3.5 Hz, 2H), 7.64 (d, J = 1.2 Hz, 2H), 6.70 (dd, J = 3.7, 1.7 Hz, 2H), 4.11 (m, 4H), 1.68 (m, 4H), 1.31 (m, 44H), 0.87 (t, J = Hz, 6H). **MALDI-TOF** (*m/z*): 661.

2,5-bis(tetradecyl)-3,6-di(5-bromofuran-2-yl)2,5-dihydropyrrolo[3,4-c] pyrrole-1,4-dione (*33*): 2,5-bis(tetradecyl)-3,6-di(furan-2-yl)2,5-dihydropyrrolo[3,4-c] pyrrole-1,4-dione (400mg, 0.606mmol) was dissolved in chloroform (45mL) and the solution shielded from light and cooled to 0°C. N-bromosuccinimide (227mg, 1.273mmol) was added to the flask portion-wise and the reaction mixture stirred at 0°C for 30 min. The ice bath was then removed and the reaction stirred for 12 hr at RT. The solvent was removed *in vacuo* and the resulting solid was purified via flash column chromatography (chloroform) to yield a dark pink solid (339mg, 68%). **^{1}H NMR** (400 MHz, $CDCl_3$) δ (ppm): 8.25 (d, J = 3.7 Hz, 2H), 6.63 (d, J = 3.7 Hz, 2H), 4.05 (t, J = 7.6 Hz, 4H), 1.69 (m, 4H), 1.41-1.22 (m, 40H), 0.87 (t, J = 6.9 Hz, 6H). **MALDI-TOF** (*m/z*): = 818.

2,5-Dibromofuran (*34*): Furan (10.0g, 147mmol) was dissolved in DMF (60mL) in a 250mL three-neck RBF equipped with a dropping funnel and the solution cooled to 0°C. 60mL of DMF was added to the dropping funnel along with bromine (46.98g, 294mmol) and this solution added drop-wise to the furan over a 30 min period with the flask open to the air. The reaction mixture was stirred for 18 hr at 15°C. The completed reaction was then poured into water (400mL). Diethyl ether (150ml) added and the layers separated. The ether layer was then extracted and washed with saturated sodium carbonate solution (50 mL) followed by saturated sodium thiosulphate solution (50 mL). The organic phase was washed once more with water (100mL) and then dried over Mg_2SO_4, before the solvent was removed *in vacuo*. The resulting oil was purified by vacuum distillation to yield a pale yellow oil (12.07g, 36%). **^{1}H NMR** (400 MHz, $CDCl_3$) δ (ppm): 6.30 (s, 2H). **LRMS** (EI+) *m/z*: 226.

2,5-Bis(trimethylstannyl)furan (*34*): 2,5-Dibromofuran (4.0g, 17.71mmol) was added to anhydrous tetrahydrofuran (60mL) and the solution cooled to -78°C. n-BuLi (22.76mL, 36.41mmol) was added drop-wise over 30 min and then stirred for a further 15 min, maintaining a temperature of -78°C, followed by 1 hr at RT. The reaction mixture was then cooled back down to 78°C to which trimethyltin chloride (7.41g, 37.19mmol) was added. The reaction was stirred for 15 min at -78°C and then overnight at RT.

Diethyl ether (100 mL) was added to the reaction mixture followed by water (150mL). The organic phase was separated, dried over Mg_2SO_4 and concentrated *in vacuo* to yield a brown oil. This oil was then purified by vacuum distillation yield a colourless oil (2.03g, 29%). ^{1}H NMR (400 MHz, Acetone-d6) δ (ppm): 6.65 (m, 2H), 0.30 (m, 18H).

C_{14}PDPPF-F: A 25mL microwave vial and stir bar were oven dried and then equipped with a rubber bung. The vial was then cooled under argon and anhydrous chlorobenzene (3 mL) added. 2,5-Bis(trimethylstannyl)furan (147mg, 0.373mmol) was then injected directly into the solvent, using the chlorobenzene to wash out the needle. 2,5-bis(tetradecyl)-3,6-di(5-bromofuran-2-yl)2,5-dihydropyrrolo[3,4-c] pyrrole-1,4-dione (306mg, 0.373mmol) was then added and washed to the bottom of the vial with a further 6 mL of chlorobenzene. The reaction mixture was then purged with argon for 20 min prior to the addition of Pd_2dba_3 (7.5mg, 0.0082mmol) and the ligand tri(o-tolyl)phosphine (10.0mg, 0.0328). The vial was then sealed and the reaction mixture degassed for 25 min with argon. The mixture was refluxed for 24 hr at 120°C. The mixture was allowed to cool and then precipitated into stirring methanol (200mL), using chloroform (3mL) to wash out any remaining product. The precipitate was filtered into a Soxhlet thimble and purified by Soxhlet extraction with acetone (12 hr) and hexane (12 hr) followed by collection in chloroform. The chloroform fraction was concentrated *in vacuo* and precipitated into stirring methanol a final time before filtration to yield a dark solid (228mg).

section S2. XRD data of C_{14}DPPF-F thin films

X-ray diffraction (XRD) measurements were carried out with a Bruker D4 Endeavour diffractometer equipped with a nickel-filtered Cu Kα1 beam and a scintillation counter detector and post-sample graphite monochromator, using a current of 30 mA and an accelerating voltage of 40 kV.

Drop-cast polymer thin films (5 mg/mL solution in chlorobenzene) show peaks at 2θ = 4.3° and 8.6° corresponding to the (100) and (200) Bragg reflections and indicative of a typical lamellar packing distance of 2.05 nm.

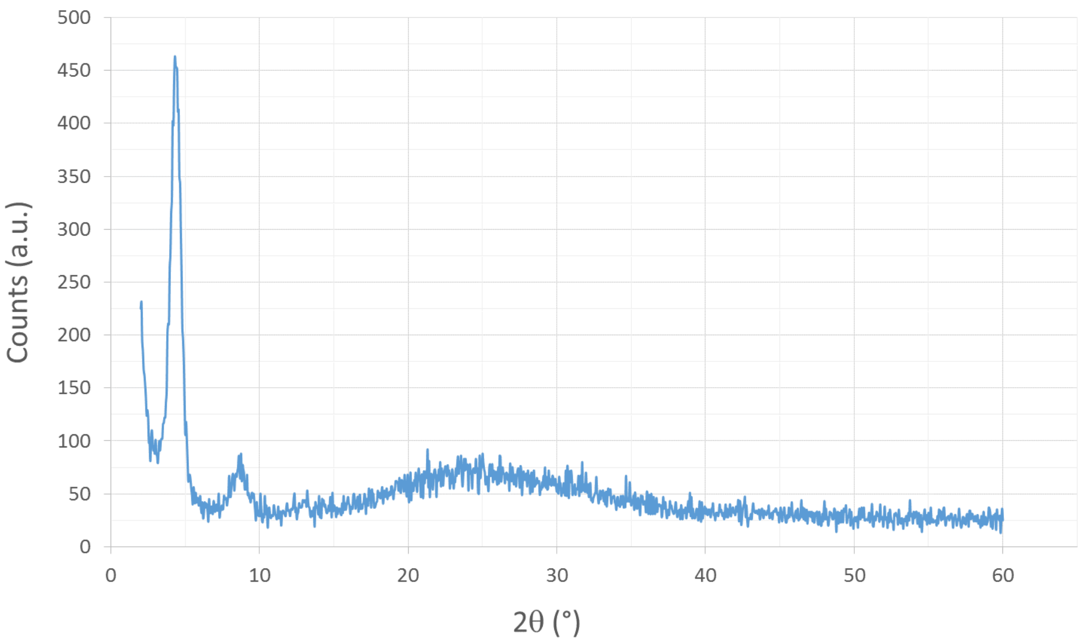

fig. S1. XRD of a drop cast thin film of C_{14}DPPF-F.

section S3. Conformation energy difference in model system via ab initio calculations

Calculations of isolated model systems (fragments or oligomers with dangling bond saturated with hydrogen atoms) have been performed to compare the energy of relative conformations and/or to compute the barrier for their interconversion. The calculations have been performed using density functional theory (DFT) at the 6-31G*/B3LYP level (analogous calculations performed recently display a very modest dependence on the basis set and the type of density functional (*35*)).

A calculation was performed to establish the local conformation of the alkyl chain when connected to the C_{14}DPPF-F polymer backbone. The model used, represented in fig. S2, contains the DPP and furane units and one alkyl chain replaced by a methyl and the other replaced by a propyl. The conformation where the alkyl chain is in the same plane of the molecule (fig. S2B) is 252 meV higher in energy than the conformation where the central carbon atom of the isopropyl is above the plane of the conjugated fragment and minimizes the repulsion with the neighbouring oxygen atom (fig. S2A). In particular, the optimal conformation has the axis of the alkyl chain oriented about 30° with respect to the DPP plane, similar to what obtained for small furan-DPP molecules (*19*). As discussed in the main manuscript, this local conformation explains the protrusion on the STM images in correspondence of the second/third carbon atom of the alkyl chain.

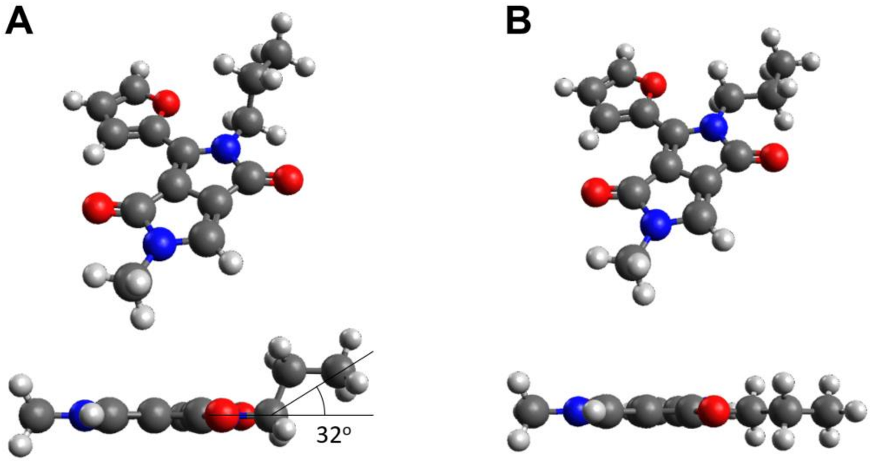

fig. S2. Ab initio calculations of the conformation of alkyl chains with respect to the polymer backbone. (**A**) Lowest energy conformation with the alkyl chain forming an angle of 32° with the plane of the DPP unit. (**B**) Conformation with the alkyl chain in the same plane as the DPP unit, 252 meV higher in energy than the conformation in (A).

As discussed in the main manuscript, the experimental images are consistent with a conformation of the polymer chain where two adjacent furane rings have the oxygen atom in cis conformation, unlike the expected conformation where all oxygens of the furane fragments are in trans relative conformation. We computed the relative energy and the barrier for interconverting between the two conformations in an oligomer, see fig. S3, finding that placing one of the furanes in a cis conformation (fig. S3B) has a modest energetic cost of 57 meV with respect to the most stable all trans conformation and that the barrier for the interconversion is 321 meV and can thus be easily overcome at room temperature. We have also computed the electronic coupling between the DPP units mediated by the tri-furane bridge in different conformation following the method introduced in Ref. (*35*) and we found that this quantity is not affected by the relative conformation of the furane bridge, changing from 158 to 160 meV when one of the furane changes to the cis conformation.

The calculations in vacuum yield a geometric structure where two consecutive DPP units are not exactly parallel as instead suggested by the experiment. To estimate the energy cost required to make the DPP units parallel, the structure was optimised by constraining the distance between nitrogen atoms in two DPP units to be identical. This straightened conformation (fig. S3C) is 564 meV higher in energy and can be justified from the improved packing of the interdigitated chain. The interaction energy between two C_{14} alkyl chains can be estimated to be in the range of 170 meV from the enthalpy of vaporization of decane (C_{10}) (*36*), where a shorter alkane was considered to account for the initial part of the actual C_{14} alkyl chain that is not parallel to the surface (see fig. S2A). For the typical sizes of C_{14}DPPF-F molecular islands observed in the experiment, this implies that the energy gain obtained by the interdigitated packing of straight polymers strands with parallel DPP units is of the same size of the energetic cost for attaining this conformation (there are two interactions per DPP monomer in the straighten polymer). As a matter of fact, straight polymer backbones are observed within the molecular islands, while polymer strands appear much more curved when in isolation (see fig. S7).

On the other hand, as already noted in the main paper, we expect a fairly strong influence of the metal substrate (not included in the calculations) on the alignment of the polymer strands. The strong polymer-substrate interaction, demonstrated for example by the observation that the backbones of the polymers are mostly aligned with the $[11\bar{2}]$ directions of the Au(111) herringbone reconstruction, and the likely

electronic hybridisation between the two could cause significant deviations from the results of the in vacuum calculations.

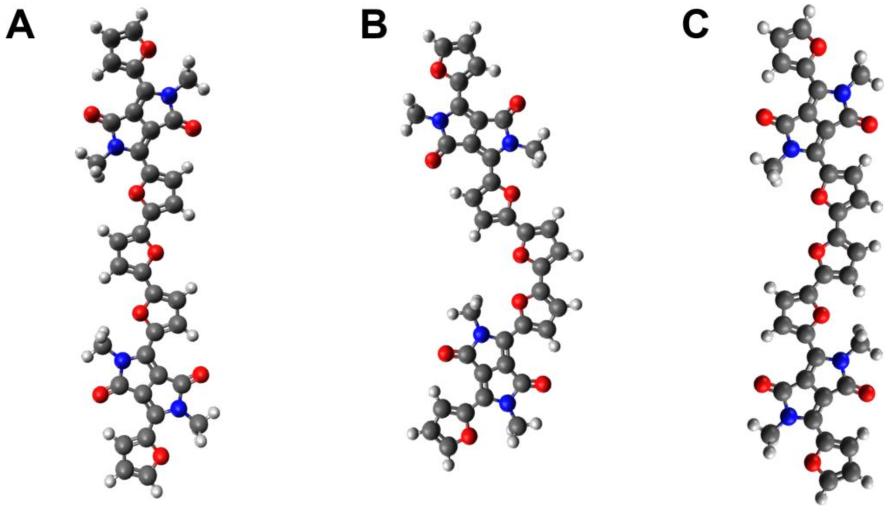

fig. S3. Gas-phase optimized structure of a C_{14}DPPF-F oligomer. (**A**) Optimized structure for the all-trans conformation. (**B**) Optimized structure with one pair of furane units in cis conformation, obtained from (A) through 180° rotation around a C-C bond. (**C**) Optimized structure obtained from (B) by constraining the DPP units to be parallel.

section S4. Assignment of A and B units in submonomeric resolved STM images of C_{14}DPPF-F

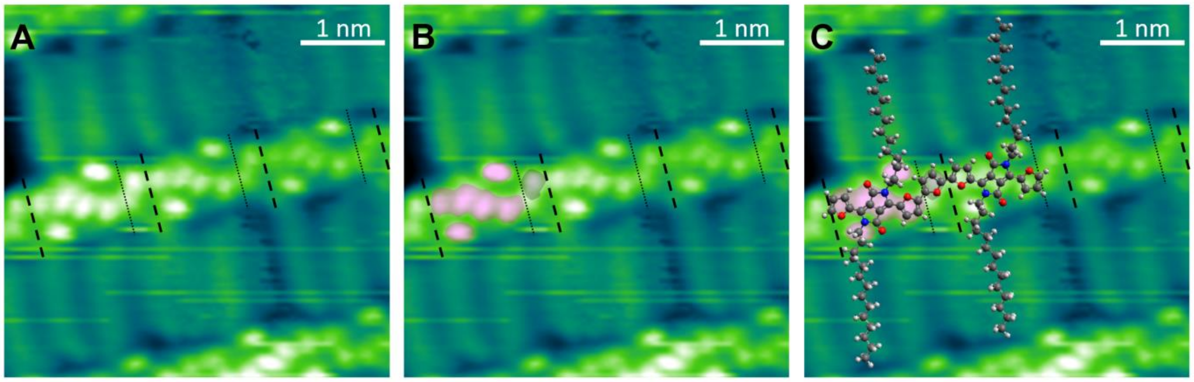

fig. S4. High-resolution STM images of C_{14}DPPF-F and corresponding molecular models. (**A**) Monomer (AB) repeat units are separated by thick dashed lines, individual A (DPPF) and B (furan) subunits by thin dotted lines. (**B**) Same image as (A) with the characteristic 4+2 bright lobes of A and the single bright lobe of B highlighted in pink and grey, respectively. (**C**) Same image as (B) with the molecular model superposed onto part of the central polymer strand. V_{bias} = –1.8 V, I = 300 pA.

section S5. SEC analysis of C_{14}DPPF-F

SEC (PS) was performed on two separate systems. Using a Shimadzu Prominence gel permeation chromatograph (GPC) with chlorobenzene at 80°C as the mobile phase, clear evidence of aggregated species was observed (fig. S5). When run using a high temperature Agilent PL220 instrument systems using 1,2,4-trichlorobenzene at 160 ^{o}C, it could be seen that the peak shape was significantly improved (fig. S6). However, the extremely broad polydispersity index (PDI) indicates that solution aggregation may still be occurring.

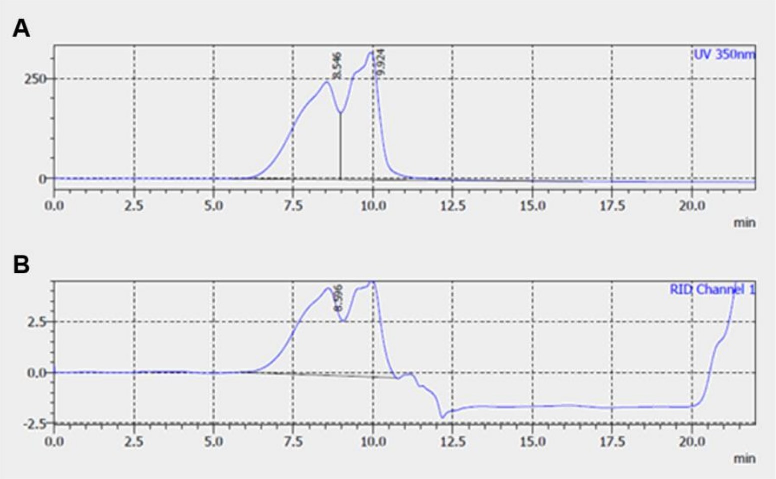

fig. S5. GPC molecular weight analysis of C_{14}DPPF-F at 80°C. (**A**) UV-Vis detector. (**B**) differential refractive index detector. Number average molecular mass, M_n = 7,265 Da; weight average molecular mass, M_w = 70,343 Da; dispersity index, Đ = 9.7.

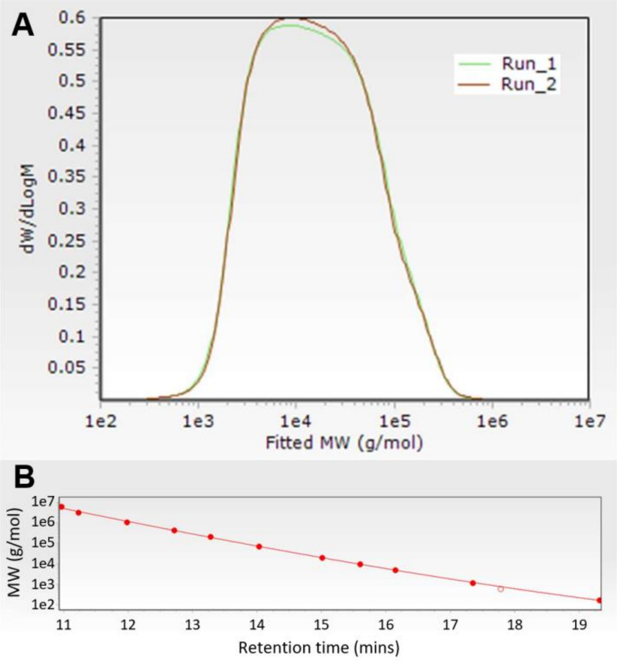

fig. S6. GPC molecular weight analysis of C_{14}DPPF-F at 160°C. (**A**) Overlay of molecular weight distributions for two repeat experiments. Run_1: M_n = 6,400 Da, M_w = 37,300 Da; Đ = 5.87. Run_2: M_n = 6,500 Da, M_w = 39,400 Da; Đ = 6.06. (**B**) Third order polystyrene conventional calibration curve between 6,035,000-163 g/mol. M_p = 108,700 (–4.1 % error).

section S6. Surface-adsorbed C_{14}DPPF-F polymer strands

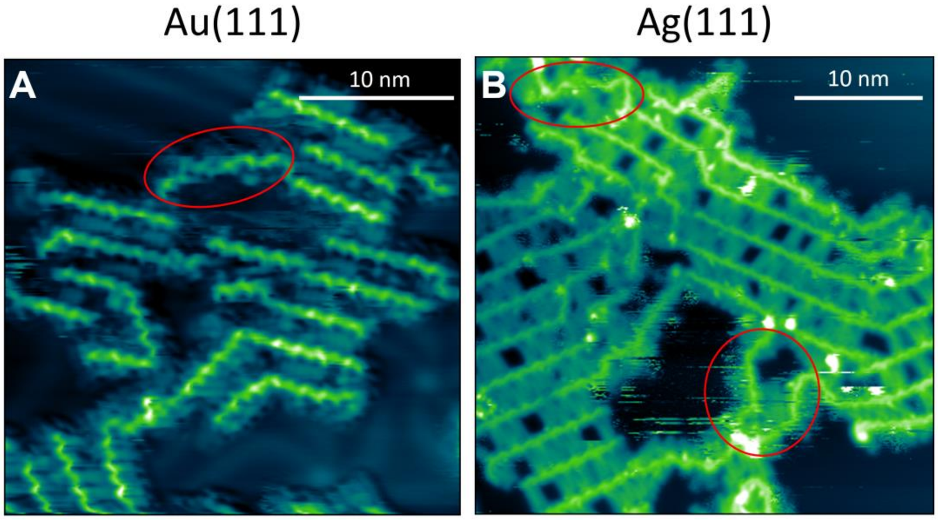

fig. S7. STM images of C_{14}DPPF-F polymers deposited on Au(111) and Ag(111). (**A**) STM image showing C_{14}DPPF-F adsorbed on Au(111) after annealing to 100°C. The polymer backbones are mostly straight within molecular islands in order to maximise side-chain interdigitation. A curved conformation is however observed for isolated strands, one of which is circled in red. V_{bias} = –1.0 V, I = 100 pA. (**B**) STM image showing C_{14}DPPF-F adsorbed on Ag(111) after annealing to 100°C. Irrespectively of the substrate, the same alkyl chain interdigitation and the same structure of the backbone (including monomer orientation and defects) as observed. In particular, also on Ag(111) interdigitated polymers have a straight backbone conformation while isolated strands are much more curved. V_{bias} = 2.0 V, I = 100 pA.

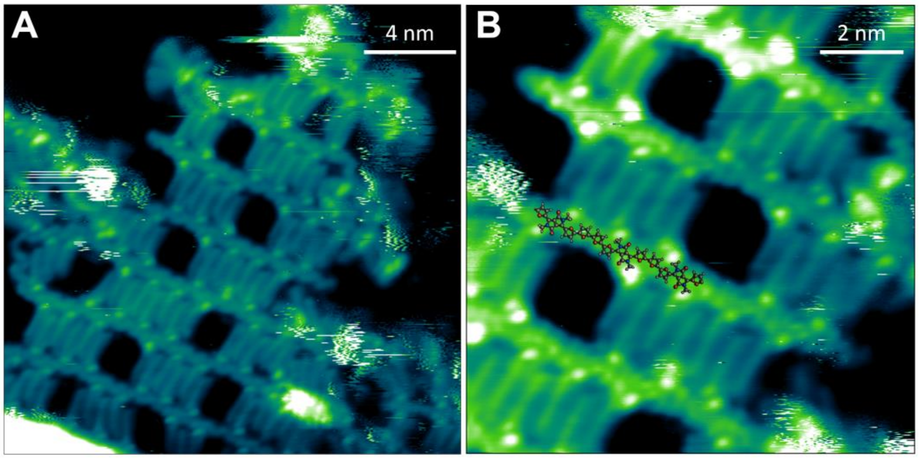

fig. S8. STM images of C_{14}DPPF-F polymers deposited on Ag(111) after annealing to 100°C. (**A**) Large scale STM image showing dark gaps in the interdigitation sequence of alkyl side-chains caused by ABBA defects in the monomer sequence. (**B**) Higher magnification STM image demonstrating the parallel orientation of DPP units in unfaulty sections of the polymer strand, and specular orientation across defects. A molecular model of the polymer backbone is overlaid on part of a polymer strand. The alkyl chains have been substituted with CH_3 groups for better visualisation. The alkyl chain interdigitation and the structure of the backbone (straightness, monomer orientation, and defects) are the same as those observed when C_{14}DPPF-F is deposited on Au(111). V_{bias} = –1.2 V, I = 200 pA.

section S7. Preliminary ESD-STM data on PDPPTPT

The PDPPTPT polymer was synthesized in the manner reported by Bijleveld *et al*. (*22*). It is a thiophene-DPP based polymer with branched alkyl chains that has shown excellent efficiencies in organic solar cells.

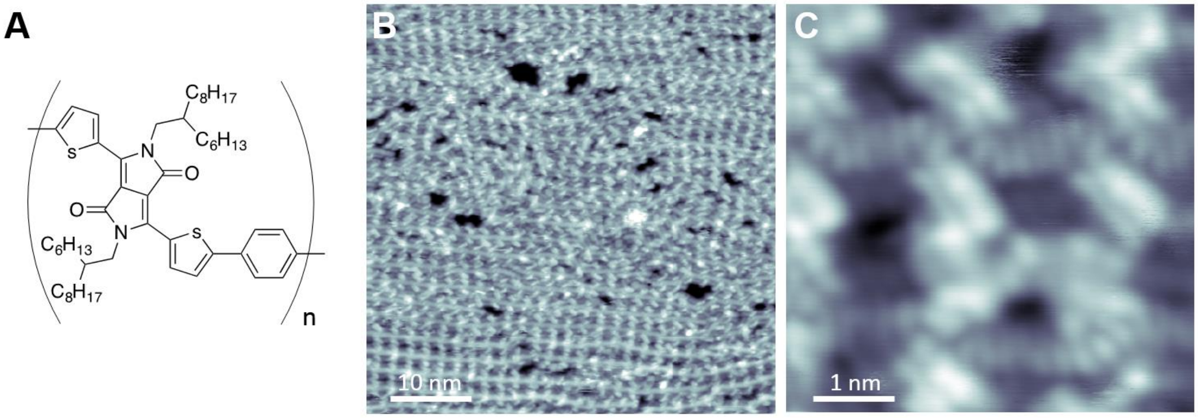

fig. S9. STM images of PDPPTPT polymers deposited on Au(111) after annealing to 100°C. (**A**) Structure of the PDPPTPT polymer, characterised by branched alkyl chains. (**B**) Large scale STM image showing the assembly of a monolayer film of PDPPTPT on Au(111). (**C**) Higher magnification STM image demonstrating sub-monomer resolution.
